# A case of HCC successfully treated with infliximab‐steroid sequential therapy for small bowel perforation due to atezolizumab/bevacizumab combination therapy

**DOI:** 10.1002/cnr2.1721

**Published:** 2022-10-12

**Authors:** Satoru Hagiwara, Yoriaki Komeda, Naoshi Nishida, Akihiro Yoshida, Masatoshi Kudo

**Affiliations:** ^1^ Department of Gastroenterology and Hepatology Kindai University Faculty of Medicine Osaka Japan

**Keywords:** atezolizumab, hepatocellular carcinoma, infliximab, intestinal perforation, steroids

## Abstract

**Background:**

Although reports of gastrointestinal perforation after immune‐related adverse events (irAE) enteritis are rare, the anti‐ vascular endothelial growth factor (VEGF) effect of bevacizumab may be involved in gastrointestinal perforation. We report a rare case of gastrointestinal perforation in a patient with hepatocellular carcinoma treated with atezolizumab/bevacizumab combination therapy and infliximab before steroid use.

**Case:**

A 72‐year‐old man, who received seven courses of atezolizumab/bevacizumab for hepatocellular carcinoma due to hepatitis B, was admitted to our department with idiopathic abdominal pain and diarrhea (grade 2 [G2]). Computed tomography (CT) and colonoscopy confirmed edema in the gastrointestinal tract. Perforation of the jejunum was observed in a CT performed on the third day and an emergency operation was performed. Intraoperative findings showed severe edema of the jejunum and leakage of feces into the abdominal cavity. The patient was diagnosed with irAE enteritis comprehensively with severe wall thickening on CT and colonoscopy, negative stool culture, and pathological findings of CD8‐positive cells. Infliximab was administered before initiating steroids, to prevent reperforation. The enteritis improved by the 22nd day; however, CT performed on the 35th day of illness showed relapse of gastrointestinal wall thickening and G2 diarrhea symptoms; therefore, prednisolone (PSL) 60 mg/day was started on the 36th day of illness. After introducing PSL, enteritis did not reoccur, and the patient was discharged on the 63rd day of illness after admission.

**Conclusion:**

There are no reports of gastrointestinal perforation by atezolizumab/bevacizumab for hepatocellular carcinoma, and prior administration of infliximab. We therefore report the clinical course and management.

## 1 INTRODUCTION

The anti‐PD‐L1 antibody, an immune checkpoint inhibitor, exerts an antitumor effect mainly by maintaining the activation of T cells by inhibiting the binding of PD‐1 and PD‐L1.^1^ Anti‐ vascular endothelial growth factor (VEGF) inhibitors have become the mainstay of cancer treatments where tumor growth is attributed to abundant neovascularization. Therefore, a combination therapy with atezolizumab and bevacizumab is considered as one of the standard treatment options for unresectable hepatocellular carcinoma (HCC).^2^

However, immune‐related adverse events (irAEs), which can develop in various organs, have become a matter of concern.^3^, ^4^, ^5^ Although reports of gastrointestinal perforation after irAE enteritis are rare, the anti‐VEGF effect of bevacizumab may be involved in gastrointestinal perforation.^6^, ^7^ Herein, we report a rare case of gastrointestinal perforation in a 72‐year‐old man with HCC being treated with atezolizumab/bevacizumab combination therapy.

## 2 CASE REPORT

A 72‐ year‐ old man was admitted to the Department of Gastroenterology and Hepatology of our institute with the chief complaints of abdominal pain and diarrhea. The patient received a total of seven courses of atezolizumab/bevacizumab from October 2021 for HCC (Child Pugh grade A; Table 1) and subsequently developed idiopathic abdominal pain and diarrhea. He was treated with intestinal regulators for several days before being admitted to our hospital. The patient had a medical history of esophageal varices (Lm,F2,Cb,RC0 → post endoscopic variceal ligation) and portal vein thrombosis with no significant familial history and no smoking and drinking habits. At the time of referral, the patient was being orally administered famotidine (40 mg/day), entecavir hydrate (0.5 mg/day), warfarin potassium (2 mg/day), rifaximin (600 mg/day), and L‐isoleucine, leucine, and valine (12.45 mg/day).

**TABLE 1:** Laboratory data on the start of atezolizumab/bevacizumab treatment

Hematology		Blood chemistry		Viral marker	
WBC	2.83/μl	TP	6.8 g/dl	HBsAg	(+)
RBC	425 × 10^4^/μl	Alb	3.5 g/dl	HBV‐DNA	(−)
Hb	14.5 g/dl	BUN	13 mg/dl	HCVAb	(−)
Hct	42.8%	Cr	0.75 mg/dl	*Tumor marker*	
Plt	5.1 × 10^4^/μl	T‐Bil	1.9 mg/dl	AFP	262 ng/ml
Neutro	62.9%	D‐Bil	0.6 mg/dl	AFP‐L3	12.3%
Lympho	25.1%	ALP	152 U/L		
Eosino	2.5%	AMY	20 U/L		
*Endocrine*	LDH	231 U/L			
TSH	3.80 μIU/mL	AST	43 U/L		
FT4	1.1 ng/dL	ALT	24 U/L		
*Coagulation*	γGTP	21 U/L			
PT	45.5%	CRP	0.124 mg/dl		
INR	1.53				

Abbreviations: AFP, α‐fetoprotein; ALT, alanine aminotransferase; AST, aspartate aminotransferase; CRP, C‐reactive protein; HBsAg, hepatitis B surface antigen; HCVAb, hepatitis C virus antibody; INR, international normalized ratio; PLT, platelet; PT, prothrombin time.

Physical examination at the time of admission revealed the following features:

height, 165 cm; weight, 59.0 kg; body temperature, 37.5°C; blood pressure, 120/70 mmHg; pulse, 90 beats/min; and respiratory rate, 16 breaths/min. No rebound tendernesss in the middle of the abdomen was noted, but the liver was palpable by two lateral fingers and lower limb edema was observed. The blood test showed a marked increase in C‐reactive protein level (Table 2). In addition, albumin and platelet count levels were decreased, indicating liver cirrhosis. The patient tested positive for hepatitis B surface antigen, but negative for hepatitis virus B DNA.

**TABLE 2:** Laboratory data on admission

Hematology		Blood chemistry		Viral marker	
WBC	6.58/μl	TP	6.6 g/dl	HBsAg	(+)
RBC	413 × 10^4^/μl	Alb	3.0 g/dl	HBV‐DNA	(−)
Hb	14.3 g/dl	BUN	25 mg/dl	HCVAb	(−)
Hct	42.7%	Cr	0.83 mg/dl	*Tumor marker*	
Plt	6.0 × 10^4^/μl	T‐Bil	3.1 mg/dl	AFP	115 ng/mL
Neutro	85.6%	D‐Bil	1.0 mg/dl	AFP‐L3	18.7%
Lympho	7.3%	ALP	151 U/L	*Immunological test*	
Eosino	0.5%	AMY	28 U/L	CD3	72.6%
*Endocrine*	LDH	281 U/L	CD4	41.1%	
TSH	3.91 μIU/ml	AST	42 U/L	CD8	27.6%
FT4	1.0 ng/dl	ALT	24 U/L		
*Coagulation*	γGTP	17 U/L			
PT	24.2%	CRP	13.298 mg/dl		
INR	2.28	PCT	3.69 ng/ml		

Abbreviations: AFP, α‐fetoprotein; ALT, alanine aminotransferase; AST, aspartate aminotransferase; CD, cluster of differentiation; CRP, C‐reactive protein; HBsAg, hepatitis B surface antigen; HCVAb, hepatitis C virus antibody; INR, international normalized ratio; PCT, procalcitonin; PLT, platelet; PT, prothrombin time.

**FIGURE 1:**
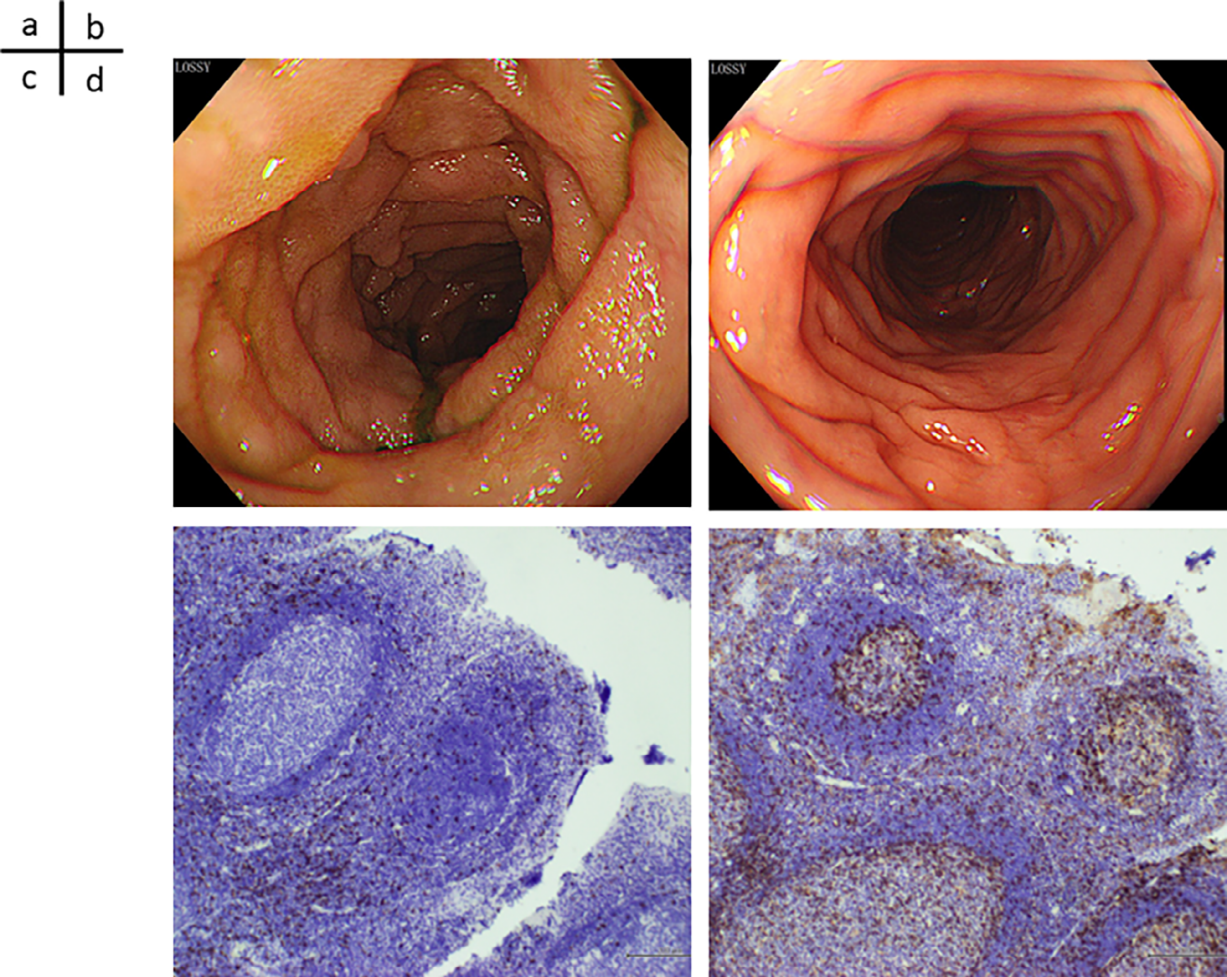
Colonoscopy and colon pathological findings. (A) Ileocecal (B) Descending colon. Colonoscopy was performed on the second day of hospitalization. Extensive edematous mucosa was found from the ileocecal region to the entire large intestine; no erosion or ulcer was noted. (C) CD4 staining (D) CD8 staining. Biopsies of the descending colon were performed. Moderate lymphocyte infiltration was observed in the subepithelial stroma, and CD8‐positive cells were also observed, suggesting immune‐related adverse events (irAE)

**FIGURE 2:**
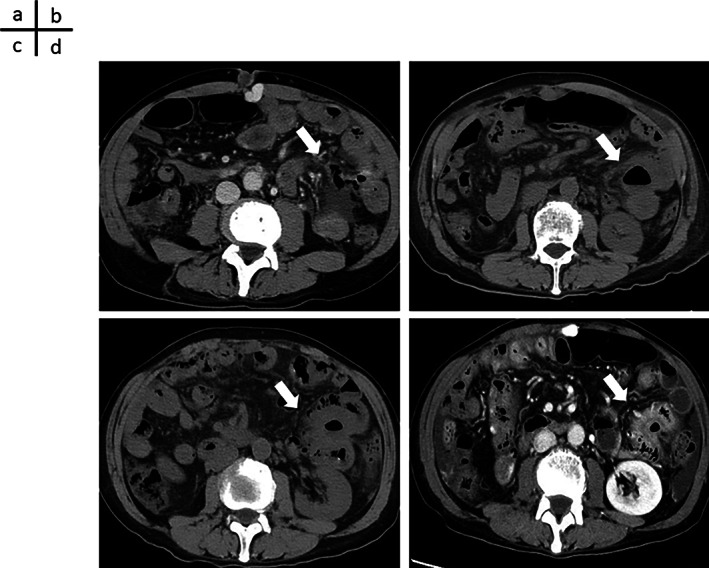
Changes in computed tomography (CT) images with the course of treatment (Arrows indicate perforated sites). (A) CT image on the third day of hospitalization. Significant edema of the jejunum and large intestine was observed. An air bubble in the mesentery and a jejunal perforation were noted. (B) CT image on the 22nd day of hospitalization (after two doses of infliximab). Improvement of gastrointestinal wall thickening and disappearance of free air was confirmed. (C) CT image on the 35th hospitalization day. Relapse of gastrointestinal wall thickening was noted. (D) CT image on the 56th day of hospitalization (after introduction of prednisolone). Improvement in gastrointestinal wall thickening was noted

**FIGURE 3:**
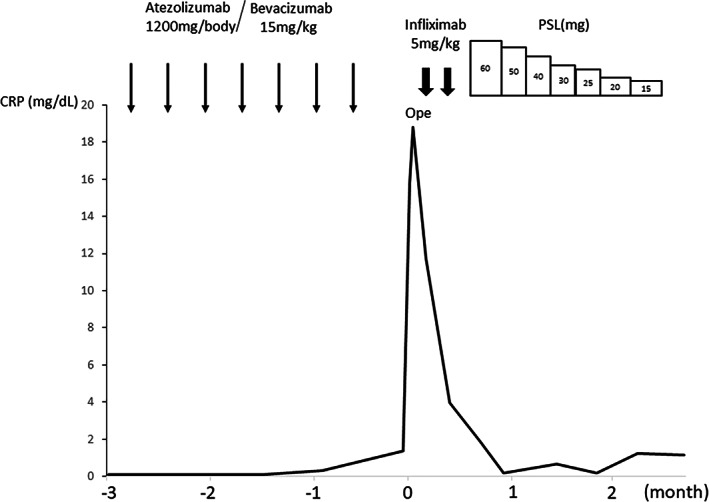
Clinical course. Seven courses of atezolizumab/bevacizumab were administered for hepatocellular carcinoma, but idiopathic abdominal pain and diarrhea (grade 2) developed. On the third day after admission, jejunal perforation was observed, and emergency surgery was performed. After two doses of infliximab at two‐week intervals, improvement of enteritis and disappearance of free air were confirmed. Thereafter, grade 2 diarrhea relapsed and the patient was administered on PSL (60 mg/day.) The patient was discharged on the 63rd day after admission. CRP, C‐reactive protein; PSL, prednisolone

Computed tomography (CT) performed on the first hospital day showed edema of the entire gastrointestinal tract. A colonoscopy performed on the second hospital day showed widespread edematous mucosa from the ileocecal region to the entire large intestine, but no erosion or ulcer was noted (Figure 1). On the third hospital day, the abdominal pain further increased, and a repeat CT showed air bubble in the mesentery and perforation of the jejunum (Figure 2); thus, an emergency surgery was performed. Intraoperative findings showed severe edema of the jejunum and leakage of feces into the abdominal cavity. However, the perforation could not be confirmed, and intraperitoneal lavage and drain placement were performed. Biopsy of the descending colon exhibited moderate lymphocyte infiltration in the subepithelial stroma, and the appearance of CD8‐positive cells, which was indicative of irAEs. A comprehensive diagnosis of irAE enteritis was made based on the course of treatment, which showed that the onset occurred after ICI administration, thickening of the intestinal wall on CT, and negative stool culture results. Although steroid treatment for irAE enteritis was considered, infliximab was administered in advance because it was immediately after perforation of the jejunum. CT performed on the 22nd hospital day, after a total of two doses of infliximab at two‐week intervals, confirmed improvement of enteritis and disappearance of free air. However, CT performed on the 35th day of illness showed relapse of gastrointestinal wall thickening and grade 2 symptoms of diarrhea appeared; therefore, administration of prednisolone (PSL) (60 mg/day) was initiated on the 36th day of illness. After the introduction of PSL, no recurrence of enteritis was observed. The CT performed on the 56th day of hospitalization showed improvement in enteritis, and the patient was discharged from the hospital on the 63rd day of illness, when PSL was gradually reduced to 15 mg/day. The clinical course of the disease has been presented in Figure 3. About 2 months have passed since discharge from the hospital, but there has been no recurrence of enteritis. Although chemotherapy was not restarted, there was no exacerbation of HCC, and quality of life was maintained. Written informed consent has been obtained from patients for the publication of case details and the use of images.

## 3 DISCUSSIONS

IrAEs is a problem peculiar to immune checkpoint inhibitors and is caused due to an autoimmune reaction. IrAEs can occur in all organs, and in addition to colitis, endocrine abnormalities, rashes, interstitial pneumonia, liver damage,^3^, ^4^, ^5^ and infrequent but fatal side effects have also been reported.^8^ However, perforation of the gastrointestinal tract is extremely rare, as in this case.^6^, ^7^ In contrast, anti‐VEGF inhibitors reportedly have a high risk of gastrointestinal perforation. Bevacizumab binds to VEGF and inhibits angiogenesis by preventing the interaction of VEGF with endothelial cell surface receptors. Although it has been investigated mainly for colorectal cancer, the incidence of gastrointestinal perforation in bevacizumab combination chemotherapy is 0.9%–3.6%, and the onset time is often within 6 months after the start of administration.^9^ Additionally, Cao et al. reported that the frequency of gastrointestinal perforation with chemotherapy without bevacizumab is 0.13% (2/1508), whereas that in the bevacizumab group is 1.0% (15/14912).^10^ The mortality rate of gastrointestinal perforation is reported to be as high as 21.5%.^9^ These reports reported the incidence of colorectal cancer, especially at the infiltration site of colorectal cancer. On the other hand, there is also a report that perforation occurred in the small intestine without cancer infiltration,^11^ and there is a possibility of perforation risk regardless of the presence or absence of cancer infiltration.

Perforation of the esophagogastric junction has also been reported in combination therapy with atezolizumab/bevacizumab.^12^ However, this case had a history of intra‐abdominal stereotactic ablative radiotherapy (SABR), which is a major difference from our case.

In this case, enteritis developed approximately 4 months after combination therapy with atezolizumab and bevacizumab, and gastrointestinal perforation occurred within a short span of 1 week after the onset of enteritis. It is also important to determine whether the causes of enteritis and gastrointestinal perforation was the use of anti‐PD‐L1 antibody or anti‐VEGF inhibitors. Infiltration of CD8‐positive lymphocytes in the endoscopic colorectal biopsy was observed despite the early onset of enteritis, suggesting irAE enteritis. However, as mentioned above, gastrointestinal perforation by anti‐PD‐L1 antibodies is extremely rare; hence, it is possible that anti‐VEGF inhibitors also had an effect. VEGF is involved in angiogenesis, which occurs during the proliferative phase of wound healing where granulation is promoted. In other words, it is highly possible that inflammation of the gastrointestinal tract and delayed wound healing due to VEGF inhibition interacted, leading to gastrointestinal perforation.

The treatment of irAE enteritis is based on guideline. The Japanese Society for Medical Oncology (JSMO) recommends that prednisolone 0.5–1 mg/day be initiated if G2 diarrhea persists for 3 days or longer. If G2 diarrhea persists for more than 1 week or if it transforms to G3 diarrhea, prednisolone 1–2 mg/day is recommended. In addition, for steroid resistance, we recommend an additional dose of infliximab (5 mg/kg) within 3 days. American Society for Medical Oncology (ASCO) and European Society for Medical Oncology (ESMO) guideline also recommend immediate addition of infliximab for steroid‐resistant cases.^13^, ^14^ In this case, gastrointestinal perforation occurred due to irAE enteritis and fecal matter leaked into the abdominal cavity. However, the perforation could not be located during surgery, and there was a concern that re‐perforation and peritonitis would develop owing to the persistence of irAE enteritis. Therefore, early intervention is essential for irAE enteritis. Although steroid administration was originally recommended for each GL, infliximab was administered prior to steroid administration in consideration of the delayed wound healing (approved by the Clinical Ethics Committee of this hospital). Infliximab administration temporarily improved enteritis, but irAE enteritis relapse was observed after two courses of administration. After confirming that there was no reperforation or infection complications during infliximab administration, steroid administration was initiated. Enteritis improvement was confirmed again, and the patient was discharged from the hospital. We believe that delaying the timing of steroid administration prior to infliximab may have contributed to the patient's recovery. In conclusion, we describe a case of small intestinal perforation caused by atezolizumab/bevacizumab combination therapy used for HCC, which was successfully managed by prior administration of infliximab before steroids. As per our knowledge, this is the first case to report gastrointestinal perforation caused due to atezolizumab/bevacizumab combination therapy used for HCC.

## AUTHOR CONTRIBUTIONS

**Satoru Hagiwara:** Conceptualization (equal); writing – original draft (equal). **Yoriaki Komeda:** Conceptualization (equal); data curation (equal). **Naoshi Nishida:** Supervision (equal). **Akihiro Yoshida:** Supervision (equal). **Masatoshi Kudo:** Supervision (equal).

## CONFLICT OF INTEREST

The authors declare no conflict of interest.

## ETHICS STATEMENT

The patient has consented to the release of this information.

## Data Availability

N/A.
